# It's classified: Classification, disability rights and Commonwealth Games

**DOI:** 10.3389/fspor.2023.1130703

**Published:** 2023-03-15

**Authors:** Nancy Quinn, Laura Misener

**Affiliations:** School of Kinesiology, Faculty of Health Sciences, Western University, London, ON, Canada

**Keywords:** parasport, sport classification, disability rights, developing nations, accessibility

## Abstract

Sport events are often held up as opportunities to showcase excellence and further access to sport participation. The ethos of accessibility has come to the forefront of many events, but none more so than the Commonwealth Games (CG). CG uses the ethos of inclusivity to bring the Commonwealth (CW) community together and utilizes sport to celebrate, uphold and drive its vision and values: Humanity, Destiny, Equality. However there remain significant gaps in participation opportunities and the realization of equality through CG, particularly for lower resource CW nations. CG is also the only global multisport event that integrates athletes with disabilities (para sport athletes), and yet there persist significant constraints to the creation of equitable opportunities for full participation for many para sport athletes. Shalala wrote “How can you effectively achieve integration (during CG), while ensuring the gulf between the best and the rest doesn't become a seismic divide?” We echo Shalala's concerns. Through this review we intend to examine sport classification as exemplary of the opportunities and hindrances for CG to actualize their values of “equality, humanity and destiny” for para sport and athletes, specifically from developing CW nations, and guard against the growing chasm “between the best and the rest”. Of significance, we consider, through a human rights lens and the concept of structural violence, the impact of sport classification on the integration of para sport and athletes at CGs, and the future of Commonwealth-wide participation and the integrated model itself.

## Introduction

The ableization of para sport, facilitated by the elimination of sport classifications for athletes with higher needs, has a “wicked” impact on para sport participation and development (1–3). The elimination of sports and events where athletes with higher needs compete have also led scholars to argue that this approach to development is antithetical to the ethos of para sport (3, 4). Yet this practice continues in the name of managing sizes of events and offering media friendly sport. Mainstreaming or ableizing para sport by “classifying out” athletes with higher needs fuels regressive social understandings of bodily difference. Conceptually, ableism locates bodies with physical, intellectual, and sensory differences as problematic, as non-normative, and imbues these bodies with notions of flawed or inferior performative abilities (5, 6). On fields of play around the globe, ableization ultimately stymies para sport participation and high-performance development. This practice is counterproductive for events such as the Commonwealth Games (CG) which seeks to provide inclusive opportunities for high performance participation. The Commonwealth sport movements provides only narrow opportunities for para sport engagement at CG, which prevents smaller and developing nations from growing para sport in-nation/territory and the opportunity of “getting to the Games” (3). There is real and substantial risk to realizing the values espoused by international governing bodies such CGF, that in turn undermines these values to rhetoric and denigrates CG's social relevance. Thus, the Commonwealth movement is doomed to fail in meeting their human rights agenda.

In this paper, we aim to demonstrate how the evolution of para sport and the practice of classification, as a form of structural violence, reinforce inequities based on ableism in the Commonwealth sport movement. This review examines sport classification as exemplary of the opportunities and hindrances for CG to actualize their values of “equality, humanity and destiny” for para sport and athletes, specifically from developing CW nations, and the impact of sport classification on the integration of para sport and athletes at CGs, and the future of Commonwealth-wide participation and the integrated model itself.

## Approach to the review: rights and positionality

Aligning with the values espoused by CGF, equality, humanity and destiny, our paper is informed by a rights-based approach to understanding and interpreting the impact of sport classification over the history of the Commonwealth Games, beginning with inclusion of a para sport in 2002. A rights-based, disability studies perspective explicitly differentiates between impairment and disability and challenges ableist assumptions of ability and bodily difference. Historically, ableist ideology has informed and continues to shape social understandings about the lives and abilities of those who live with physical difference. Our approach to this paper explicitly resists, challenges, and intends to disrupt notions that pathologize difference. We are committed to understanding how to support full and equitable participation of para sport athletes in the integrated sporting space (7) and in the broader social world. We consider the processes of classification and resultant inclusion of some athletes with impairment, and subsequent exclusion of others from participation at CG as demonstrative of the ableization of Commonwealth sport (8).

We wish to acknowledge our own positionality and the potential of our subjectivity to impact our interpretation of the information reviewed in this paper. We do not live with impairment and neither of us are currently high-performance athletes. The first author has a sustained relationship with para and Paralympic sport, in Canada and abroad. Her presence at the two most recent Commonwealth Games, Commonwealth Games XXI and XXII and her commitment to disruption of ableist ideologies around sport and ability positions her well to conduct research in this space (9). The second author has extensive research experience in para and Paralympic sport spaces and has interrogated the opportunities and constraints of diverse models for sport (10, 11). She too has attended multiple para sport events, including Commonwealth Games (CG) in Glasgow, 2014 and CG held in Birmingham in 2022 (B2022). We are cognizant of our power and privilege as academics and in the case of the first author, as a health care professional. By grounding this review in a rights approach to disability, our intention is to represent our findings in a credible and meaningful manner. We believe too that the embodied experience of disability is central to the lives of para sport athletes, and that disability is the product of social and political power relations. As DePauw (12) argued, “the lens of disability allows us to make problematic the socially constructed nature of sport and once we have done so, opens us to alternative construction, actions, and solutions” (p. 428).

## Data and approach to database review

For the purposes of this review, we drew upon a large database that represented para sport inclusion at the five integrated Commonwealth Games, beginning with Manchester 2002 and including the most recent iteration hosted in Birmingham UK in 2022. The initial database was constructed as part of a para sport capacity assessment conducted by the authors in the region of the Americas and the Caribbean (11). At its inception, the database included information around para sport concerning the 20-member Commonwealth nations within the Caribbean and Americas region. The information included the major sport governing bodies in the region, publicly available contact information of key sport executives, existing and historical sport development projects, associations who support people with disabilities, post-secondary institutions with sport science curriculum, and details concerning other relevant regional organizations. The database served as a starting point for understanding the landscape of para-sport in the region and within the broader Commonwealth. CGF shared para sport information specific to CG 2002–2022, which was added to the original database. Games-specific data included names, gender, sport, event, and classification of athletes specific to each of the five CG. The sport classifications selected for each CG were identified, with associated gender, i.e., T54 male. Participating CW nations/territories with para sport team members were included in the database as well as the sport/events in which athletes from a given nation/territory competed.

Review of the database was iterative, dynamic and is ongoing. Follow up work not included in this review offered multiple semi-structured interviews to further extend on the findings of this review. For this piece, we focus on the *ad hoc* conversations that took place between authors, CGF administration, and other para sport stakeholders in the development of the database. The review was used to identify opportunities and constraints specific to the impact of sport classification across these CG. Our findings are considered in light of the historical context and commitment of inclusion of para athletes by CGF at CG, and the ongoing constraint for involvement of developing countries in the CG para sport program.

We argue that poor representation of Commonwealth nations/territories in para sport competition is indicative of persistent inequities based on ableism in the Commonwealth sport movement.

## A brief history of the commonwealth games

The Modern Commonwealth is an association of 56 member states, who as members of the Commonwealth (CW) are committed to “work together for prosperity, democracy and peace” for all nations, territories, and citizens of the Commonwealth (13, para 1). Commonwealth Sport also known as Commonwealth Games Federation (CGF) is responsible for the direction and control of the Commonwealth Games and Commonwealth Youth Games, and for delivering on the vision of the Commonwealth Sports Movement: to build peaceful, sustainable, and prosperous communities globally by inspiring Commonwealth Athletes to drive the impact and ambition of all Commonwealth Citizens through Sport (13, para 1).

The Inter-Empire Sports Championships took place in 1911 to honour the coronation of King George V and involved four competing nations including Canada, Australasia, South Africa, and England. The success of the event was the catalyst for the creation of the Commonwealth Games Federation (CGF) who would plan, oversee, and administer future, multi-sport events, striving for broad athlete representation from nations belonging to the Commonwealth (13). The British Empire Games, a subsequent iteration of what would become the Commonwealth Games (CG) took place in 1930 hosted by the city of Hamilton, Canada. Since 1978, the Games have been known as the Commonwealth Games are held on the quadrennial and are a development Games that utilize sport to celebrate, uphold and drive the vision and values of the CGF and the broader Commonwealth movement (13). Of note, the Commonwealth Paraplegic Games, also referred to as the Paraplegic Empire Games and British Paraplegic Games were hosted by CW nations from 1962 until 1974. Considered a precursor to the modern Paralympic Games, these Games preceded mainstream CG, providing competition opportunities for athletes with disabilities from CW nations. In 1974, the Commonwealth Paraplegic Games were discontinued due to insufficient financial resources and organizational capacity (14).

In a bold and vanguard move to embody these values, CGF adopted an integrated model for CG where para sport athletes from participating nations enjoy the same rights and responsibilities of competition as their mainstream teammates. Since 2002, para and non-para events belong to a single competition schedule. Medals won by para sport athletes of each nation/territory are afforded full medal status and included in the all-important medal count. As full team members, para sport athletes, coaches, technical and sport science personnel, “eat, sleep and compete” alongside their non-disabled counterparts. Informed by the values espoused by the Modern Commonwealth and their commitment to inclusion and integration in and through sport, CGF has been at the forefront of integrated, multi-sport practices and events.

At the time of writing, CG XXII (Birmingham, UK) referred to as B2022 have just closed. In her speech at Closing Ceremonies, Dame Louise Martin, President of CGF delivered heartfelt praise for the event.

*These Games have been bold, buzzing and absolutely brilliant*.
*We have seen more Para sport than ever before*.
*3 × 3 basketball and women’s T20 cricket for the very first time*.
*We have seen more medals for women than men*.

Her words are significant and important as one of the first post-Covid multi-sport events to return. B2022, and the city and venues did “buzz” with wonderful energy and included 136 medal events for athletes who identify as female and 134 those who identify as male. 3 × 3 basketball for standing and wheelchair athletes, made its debut, to the delight of fans with thrilling matches and explosive finals (15). Inclusion of the T20 women's only cricket tournament held at the venerable Edgbaston Cricket Grounds celebrated the return of the sport to CG after a 20+ year hiatus, and the unique T20 format (16). As most Games now espouse, B2022 hosted the largest para sport program since the adoption of the integrated model in 2002. The para sport program included eight para sports and the debut of 3 × 3 wheelchair basketball, upping the number of para sport events by one over CG2018. B2022 included 43 para sport events in contrast with 36 events at Gold Coast, and total of 386 para sport athletes participated, a notable increase from 251 at Gold Coast 2018 (2).

By the numbers, it appears that CGF has much to celebrate. A growing number of para sports at CG, more para sport events, and steadily more para sport participation at each iteration of the CG. However, to contextualize these numbers and the enthusiasm around the success of B2022, the numbers also indicate only 31 of 72 CW nations/territories who attended B2022 had para sport athletes on their teams. The 386 para sport athletes who competed in Birmingham comprised just 7.6% of the total athlete delegation. Of note, 86% of those participating para sport athletes came from just 10 nations/territories—England, Northern Ireland, Scotland, Wales, Australia, Canada, New Zealand, South Africa, India, and Nigeria. Over the history of the modern Commonwealth Games including B2022, these same nations/territories continue to dominate the all-time Commonwealth medal tallies (17).

## Ableism, classification, and integration

To better understand the importance of the Games by the numbers and consider the impact of sport classification on para sport participation at Commonwealth Games, we offer a short description of three important concepts that are integral to this work- ableism, classification, and integration in sport.

We begin with the concept of ableism, due in part to its social pervasiveness and persistent impact on para sport development and the athlete experience of para sport (18–20). Ableism and ableist ideology inform the theory and practices around para sport and integration in sport (3, 21, 22). Ableism describes a set of social assumptions and practices in which the non-disabled experience and perspective are dominant, normalized and socially valued (23). Ableist ideology considers able-bodiedness as natural and normal, resulting in structures, spaces and practices that emphasize this normative perspective. Because of its historic and insidious nature, ableism like other forms of systemic marginalization, like racism, sexism, and ageism, falls under societal radar. Overt and more subtle forms of ableism, exist in sport and in fact in sport medicine (2, 5, 24, 25). As identified by retired Paralympic, Howe (26) noted that more nuanced forms of ableism, subtle forms of prejudice “confronts me and those like me daily” (p. 6).

Sherrill (27) defined sport classification, as an “ever-evolving program and system that strives to make competition equitable and fair” (p. 210). Tweedy and Vanlandewijck (28) summarized the desired effects of sport classification as follows; “classification in sport reduces the likelihood of one-sided competition” (p. 3), is essential to fair competition, and should have a positive effect on rates of participation. All para sport athletes require classification to compete. Historically, the organizational structure of disability sport classification, has been medically based. Athletes with impairment needed to classified in disability or para sport, “organized into smaller entities based on observable properties that they had in common” (29, p. 3) which was a shared medical diagnosis. At one time, athletes with like-medical diagnoses competed within a diagnostically homogenous field, and an athlete's classification applied across sports. The importance of athlete classification escalated in the 1980s because of the growing competitiveness of para sport (29). Increased competition, commodification, heightened media attention, and for a few, status as an elite athlete, placed tremendous pressure on sport classification systems to evolve (29, 30). Classification, once a simple formula based on medical diagnosis, evolved to become a system informed by function rather than diagnosis. Functional classification attempts to provide an athlete with a classification by determining the impact that bodily impairment has on sporting performance. An athlete's classification is sport specific, and substantially impacts their opportunities to compete and to develop as a high-performance athlete. It is noteworthy that athletes with visual impairment (VI) currently continue to be classified by medical assessment of visual acuity (31).

**Figure 1:**
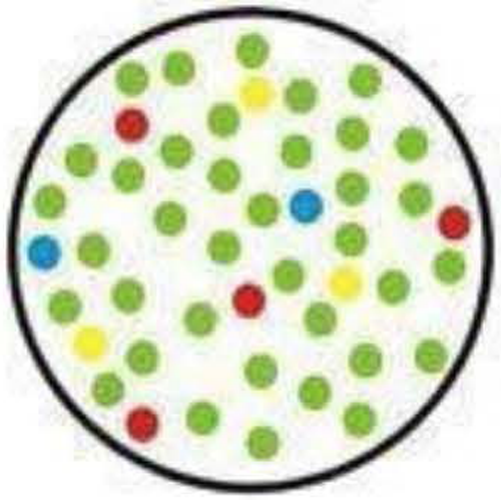
Integration in sport. Figure 1 was produced by think inclusive by MCIE as part of a schematic that reflects possible outcomes of integrative and inclusive practices in education.

The terms integration and inclusion continue to create confusion. In the sport studies literature these terms have often been used synonymously and interchangeably. For our purposes and in this work, we conceptualized integration in sport as the “intermixing of peoples previously segregated” (4, p. 148) where reciprocal adaptation of both sporting groups results (32). Inclusion refers to allowing different groups to come together but not necessarily intermixing groups. That is left up to the devices of the individuals. Figure 1 provides a visual representation of our understanding of integration in sport and underpinned this review of the impact of classification on para sport across multiple CG.

Sport organizations like CGF have adopted the term integration to mean inclusion of *some* para sports, *some* events, and *some* athletes. Quinn, Misener and Howe (3) have argued that classification has been used as a facilitator of “integration for some and not for all”. Pullen, Jackson and Silk (2) illustrated that when Paralympic teams are dominated by “abled-disabled” athletes, the absence of athletes with higher needs creates an inauthentic representation of a nation and of the Paralympic movement. Integration in sport remains a priority at many levels and for many reasons. Misener and Molloy (10) outlined the potential opportunities and constraints surrounding integration of high performance able-bodied and para sport, namely the integration of the Olympic and Paralympic Games. Practically, integration should promise maximization of Games related resources, both economic and human, and simplification of Games planning, organization, and implementation. However, integration also poses a risk to the distinctiveness of para sport and to participation of *some* athletes. Without vision, long term commitment and equitable resource allocation, assimilation rather than integration of the Paralympic Games with the Olympic Games would be the foreseeable outcome (3, 4).

Our review draws from a substantial database of the Games-specific information offering unique insights into our question about the impact of classification on para sport participation since the early days of integration (2002–2020). We raise additional questions around selection of specific para sports per CG, why events were selected/not selected within each sport, and about choices made to include/exclude classification categories at each CG. Each of these questions begs further scholarly investigation. That said, we identified several opportunities and constraints for the process of sport classification across these 20 years and considered how para sport participation has been impacted, particularly from the perspective of commonwealth small island and developing nations.

## Opportunities: moving para sport forward *via* CG

There has been steady and incremental growth in the small number of para sports selected for inclusion throughout the two decades of integrated CGs. In 2002 at Manchester UK, five sports were included on the competition schedule which included athletics, swimming, lawn bowls, table tennis and powerlifting. The following two iterations of CG, 2006 and 2010, four of these five para sports were included, with lawn bowls dropped from the competition schedules. Subsequent CGs saw the addition of a single sport in 2014 (para track cycling) and para triathlon and lawn bowls were added in 2018 (total 7 para sports). B2022 hailed the largest number of para sports on a CG's program, hosting the debut of 3 × 3 wheelchair basketball (33).

**Figure 2:**
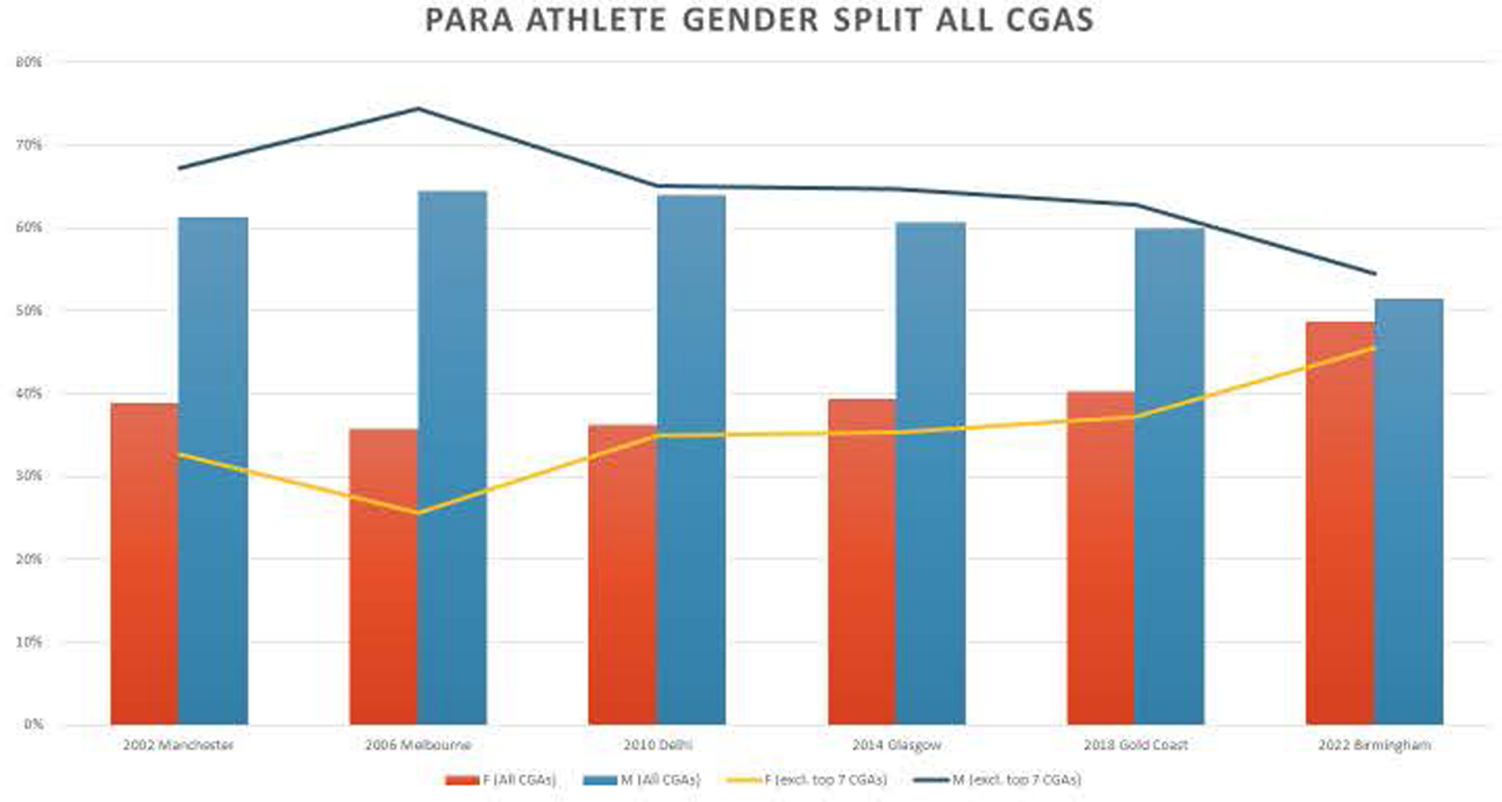
Para athlete gender parity at CG 2002-2022. Figure 2 was prepared by CGF post B2022. Pam sport analysis: for the purposes of understanding the impact of GAPS on Birmingham 2022.

A second positive trend is with respect to gender parity in para sport across the six CG (2002–2022). Review of the gender data from Manchester UK (2002) to Gold Coast AUS (2018), demonstrated persistent and significant disparity in gender representation in para sport athlete delegation. Athletes who identified as male, outnumbered those who identified as female across five CG. With equal number of events for male and female para sport athletes on the competition schedules, only 36% (average across 5 CG) identified as female, roughly one female para sport athlete for every three male athletes. Remarkably and somewhat to our surprise, this inequitable trend was disrupted at B2022 where an almost equal number of para sport athletes identified as male (51%), and female (49%) participated. As illustrated in Figure 2, the realization of gender parity at B2022 may be the result of a subtle shift in team composition of the seven historically dominant Commonwealth Games Assemblies (CGA) (Australia, Canada, England, New Zealand, Republic of South Africa, Scotland, and Wales). Going forward, we will refer to these seven, very dominant CGA as The Big Seven.

**Figure 3:**
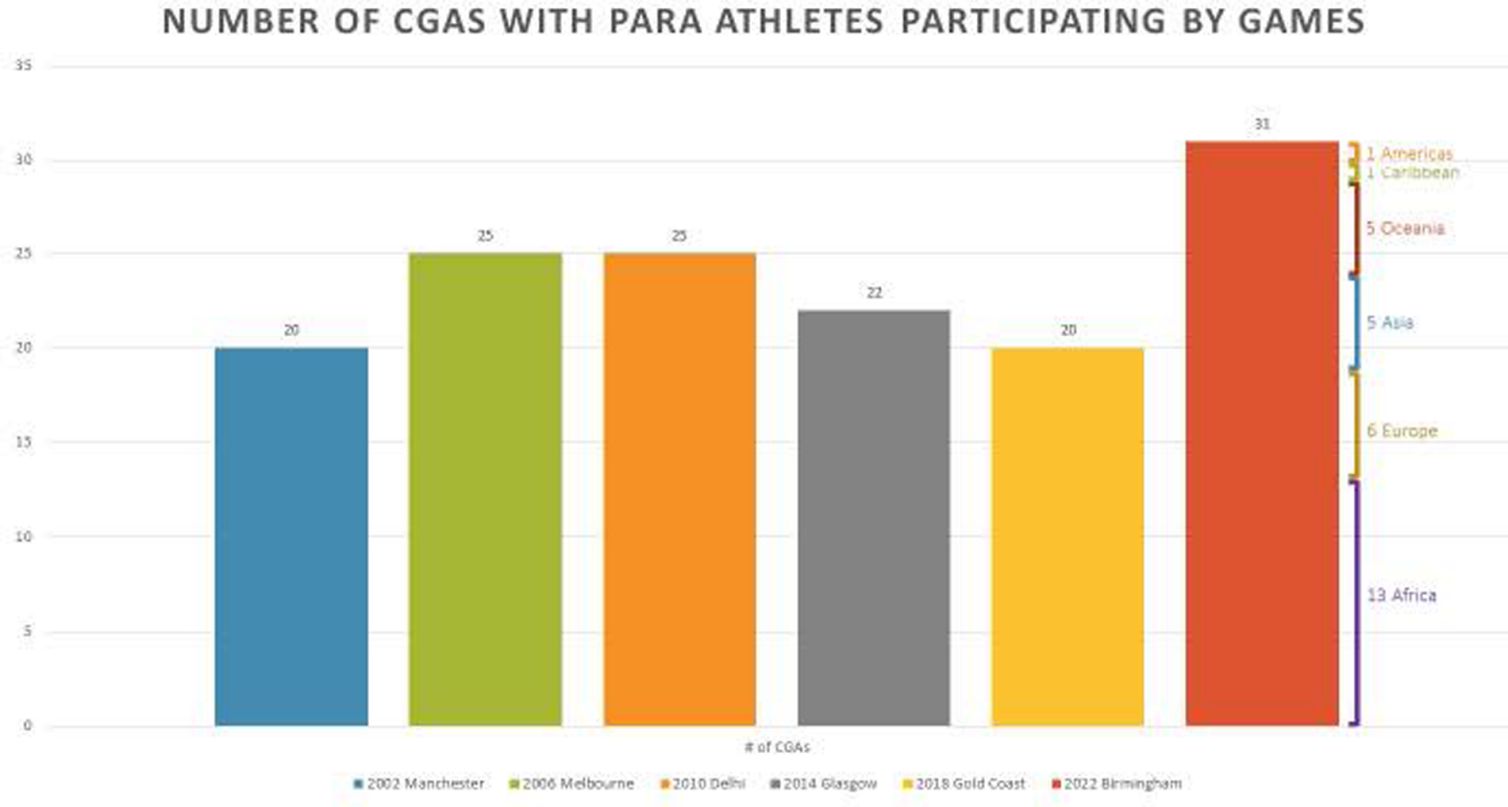
CGA's with para sport representation at CG 2002 to 2022. Figure 3 was prepared by CGF post B2022 titled pam sport analysis: for the purposes of understanding the impact of GAPS on Birmingham 2022.

Our review revealed another potentially promising opportunity regarding the valuing of para sport at CG. Across the six CG, those nations/territories who have included para sport athletes as part of their teams, continue to do so, reflecting perhaps a commitment to para sport representation. But as seen in Figure 3, this group of para sport inclusive nations and territories is small, and not representative of the broader commonwealth of developing and small island members. However, at B2022, there was a material increase in the number of delegations with para sport athletes. At B2022, 31 nation/territories included para sport athletes as members of their delegation, in contrast with 20 who did the same at Gold Coast (2018). This increase in delegations with para sport athlete representation was the result six additional African nations/territories, two European teams and three from Asia that all sent para athletes.

## Constraints: negative influences of para sport *via* CG

Quinn, Misener, and Howe (3) argued that “size does matter” and drew attention to the very small relative size of the para sport delegation at CG2018. Of the 72 CGAs in attendance at the Games, 20 delegations included parasport athletes, equal to approximate 27.7% of the total. Of the 4,426 athletes who competed at Gold Coast, 251 of these athletes competed in para sport events, 5.7% of the total athlete population. CW2022 with a total of 72 participating nations/territories, an athlete population of 5, 054, was host to 386 para sport athletes. Though marginally greater than 2018, the para sport delegation comprised only 7.6% of athletes at these Games.

**Figure 4:**
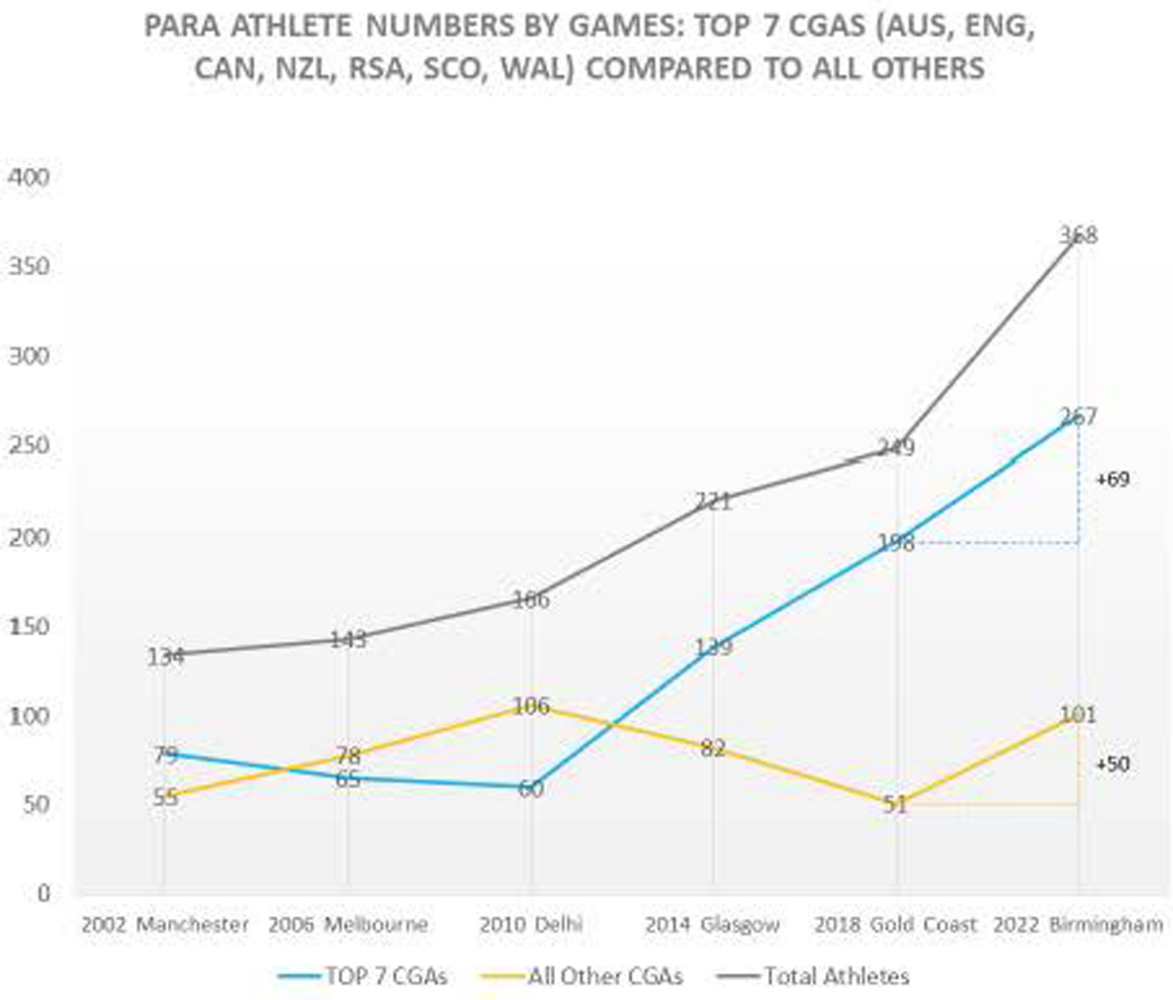
Pam sport athletes across CG: the big seven relative to other CGAs. Figure 4 was prepared by CGF post B2022 titled para sport analysis: for the purposes of understanding the impact of GAPS on Birmingham 2022.

Figure 4 below illustrates the hyper-representation of para sport athletes from The Big Seven CGAs over the six CGs at which the integrated model for competition was implemented. Perhaps more problematic than this historical reality, is the contemporary, growing gap of para sport participation of athletes from The Big Seven and other CGAs.

We return to the work of Shalala (2) and reiterate that 86% of participating para sport athletes at these Games came from just 10 nations/territories, namely The Big Seven plus Northern Ireland, India, and Nigeria. Over the history of CG including B2022, these same nations/territories have dominated the all-time medal tallies (15) and five are economic powerhouses, with the largest economies across the Commonwealth (34). The numbers tell a different story where equity of access is not part of the equation.

We return to our central argument that access is not equitable for all in the parasport movement. We argue that sport classification has a material impact on the para sport integration at CGs 2002–2022. Given the integral reality of classification to para sport participation, we offer some insights about the impact on para sport participation and evolution within the framework of the six CG. To demonstrate the inequities, we review classification within the sport of athletics, due in part to its inclusion across all Games and its potential for development in developing CW nations/territories. In 2002, the debut of the integrated competition model for CG, elite athletes with disabilities (EAD) competed in several demonstration events, including athletics, swimming (4 events each), lawn bowls, para powerlifting, and table tennis. Athletics and swimming have been part of the para sport competition for each subsequent CG, with an increasing number of events in each sport. As mentioned previously, the requisite skills associated with many athletics events are universal—run/wheel, jump, throw. In contrast with other para sport events, equipment and technology required for training and competition are minimal. CGF has been vocal about the rights of athletes from developing CW nations/territories to be represented at future CG. CGF is also acutely aware of the constraints faced by some para athletes from developing CW nations and see athletics as a point of entry (2, 35).

Our review of classifications at CG 2002–2022 revealed several insights. In 2002, four para-athletics events took place. This number doubled by 2022 for a total of nine events. In 2002, there were four medals albeit, demonstration medals awarded in para-athletics. Twenty years and five CG later, a total of 24 medals were awarded. However, as seen in the figure below, athletes from The Big Seven CGAs continue to dominate participation in athletics at all six CG. In fact, at B2022, 75% these para athletes were from one of these seven nations/territories.

There was also marked variability of classification categories from one CG to the next, including the inclusion/exclusion of classifications for male and females at consecutive CG. For example, javelin throw for females (classification F46) took place at Gold Coast 2018 but was excluded from the schedule in 2022. In 2018 shot put for male athletes (classification F38) was included on the para sport schedule. Four years later, male athletes were excluded from participation in this event and female athletes (classification F57) competed in shot put. At CG2018 and CG2022, 100 and 1,500 m track events for both male and female athletes took place. However, competition opportunities for female athletes to participate in the 100 m in 2018 and again in 2022, were substantially impacted. In 2018, 100 m female athletes with a T35 classification participated and were excluded 4 years later, due to change in classification to that of T34.

According to the International Paralympic Committee (IPC), the classifications of both T35 and T34 are one of seven athletics classifications for athletes with cerebral palsy represented alphanumerically from T32, T33, T34, T35, T36, T37 and T38 (ipc.org/classification). A lower number, in this case T34 relative to 35, indicates an athlete whose impairment has less impact on their athletic abilities. It is possible that a female athlete with a T35 classification who competed in 2018 would be “classified out” of competition in 2022, due to decisions made by CGF and the organizing committee to include sprinters with so-called lesser impairment. In other words, athletes who look more “able bodied” and require fewer adaptations. Variability of classification categories between subsequent CG is a material deterrent to para sport participation and development (18, 27), the evolution of high performance (3, 29) and to broadening representation in para sport events at future CG (3). Quinn and colleagues (3) along with other scholars (22, 29, 36) stated that elimination of sport classifications for athletes with higher support needs, or alternatively stated, athletes with more severe impairment, results in the ableization of para sport competition at CG. Integration at the Commonwealth Games translates to “integration for some and not for all” (3). We argue that this tension around integration and ableization of para sport is irreconcilable with the values espoused by CGF and their commitment to integration in and through sport.

Athletes with a T34 and T35 classification compete on the track using a race wheelchair (ipc.org/classification), the cost of which prohibits participation for athletes from lower resource nations/territories. At B2022, the six female athletes suing race wheelchairs in the 100 m event (T34) represented two nations only, three each from Australia and England. At these same Games, only four athletes participated in the women's marathon, representing only two CGA's, once again Australia and England. Due in part to the inclusion of wheelchair racing events as demonstration events at Manchester 2002, wheelchair racing remains synonymous with para-athletics. Yet, wheelchair racing events, like 100 m for T35 and T35 athletes, requires access to expensive equipment, least of all the wheelchair itself. The inclusion of para triathlon at CG 2018 and again in 2022, is further example of sport selection that excludes para sport athletes from lower resource locations. The resources required to purchase and access the technical expertise to maintain high-tech sporting equipment is beyond the means of many Commonwealth countries, with the exception of The Big Seven. As well, those who use wheelchairs for daily transportation and to complete necessary, everyday tasks report difficulties accessing public transportation, and generalized fear, a vulnerability of being in some spaces (35, 37). These same issues confront wheelchair athletes from developing Commonwealth nations/territories. Para athletics has long been considered key to para sport development, the evolution of high-performance para athletes, and “getting more athletes to the Games” from the developing Commonwealth. CGF is aware of some of the constraints around growing representation across the Commonwealth at these Games (2). Discussions with CGF leadership following CG2022 supported Shalala's comments (2). CGF also presented an argument that inclusion of the athletics classification T38 hinders participation and para athletic development in developing Commonwealth nations. Athletes who are likely to be classified as T38 are unlikely to be reside in the developing world because of limited representation of this type of impairment in these locations. Therefore, a decision to include athletes classified as T38 is a decision to include athletes from developed countries in contrast with those from lower resource nations/territories. CGF argued that that individuals with greater impairment, like would-be T37 athletes, are more likely to reside in developing CW nations/territories and that sufficient T37 athletes exist across the Commonwealth create a competitive field. CGF considered the inclusion of this classification, T37 in contrast with T38, as viable way to broaden and diversity para sport representation across the Commonwealth.

## Discussion: classification as a catalyst for para sport growth at CG

We begin by being explicit about the volume of data available for consideration about the para sport agenda of the past six Commonwealth Games. Because of the plethora of data readily available but under-analyzed, we were required to frequently return to the research question that being, what historical trajectory has sport classification had on the integration of para sport and athletes at CGs. Classification remains an integral part of high-performance para sport, with the intention of increasing participation and leveling the competitive para sport playing fields (22, 28, 29). Our review across six CG illustrated that classification can be a mechanism by which para sport participation is negatively impacted, resulting in the exclusion of athletes from lower resource nations and those with higher support needs. The research demonstrates that inclusion of some classification categories and resultant exclusion of other categories has had a negative impact on para sport representation for some CGA's and hindered para sport development in other locations. As a development Games, a sporting event committed to the values of “humanity, destiny and equality”, CG is well located to be *the* competition space for newcomers to para sport, developing para athletes, and a steppingstone for those on the elite para sport pathway. For para sport athletes involved in non-IPC sanctioned sports, like the sport of lawn bowls, CG can provide an opportunity to compete with the global best at a large scale, global sporting event, beyond that of a world championship. Many developing Commonwealth nations/territories lack the resources, infrastructure, and technical expertise to host sufficient and necessary in-nation, in-region competitions that expose athletes and coaches to the rigors of sport, experiences that are essential to athlete/coach development and maturation. CG can also be a sporting site for para athletes and coaches from smaller and lower resource nations space to develop as high-performers *via* participation, when competitions are too few and too resource-rich in their home nations.

Our review provides evidence of a number of disturbing trends around the use of classification, to include some and exclude others, particularly para sport athletes with higher support needs and fewer economic resources. Selection of sport classifications and para sport events varies from one CG to the next. An outcome of this lability of inclusion/exclusion between Games is a persistent tension around what sports, classifications and therefore para sport athletes are deemed legitimate for a given CG. The ethnographic work of Quinn, Misener and Howe (3) foregrounded the para sport athlete experience of inclusion/exclusion by classification at Gold Coast 2018, with athletes expressing anger and frustration of the inclusion/exclusion of sport classifications at each CG. Athletes spoke of the cascade effect of these decisions on their athletic success, competitive future, and the subsequent social and financial costs borne by the athletes themselves. These finding undermine CGF's ability to realize their values, namely that of equity of opportunity on the field of play for all citizens of the Commonwealth. We contend that classification is a potential tool to deny some athletes with disabilities their right, as laid out in the United Nations’ Convention on the Rights of Persons with Disabilities (38) to participate in Commonwealth sport. We suggest that classification can used as a form of structural violence, whereby athlete access to sporting participation is denied by the ableist social structures associated with classification and the with institutions control. First introduced by Galtung (39) in his peace studies research, structural violence describes a form of violence, explicit and more nuanced, whereby people are denied their human rights. As a result of this form of violence, these people experience marginalization, are limited in their right to development, and are denied social justice (40). Conceptually, structural violence is closely tied to that of social justice and emancipatory practices (39–41) and this form of marginalization is realized most by those who occupy the bottom rungs of the social ladder. Farmer and colleagues (42) described structural violence as a denial of the human rights of some brought about by “the social arrangements that put people and populations in harm's way” (p. 1686). Our review illustrates that the changing classifications between games, an institutional practice that is mandatory for para sport competition, is directly impacting para athletes’ participation in the Games, uniquely so athletes with greater impairment and those from developing CW nations/territories. This has a direct effect on their right to pursue emancipation and social equality through and in their sport when many continue to be excluded from participation.

A critical issue for CGF and many lower resource CW nations is the development of sustainable para sport pathways which should lead to broader representation at CG in para sport events and disrupt the historic dominance of a few. Games to Games flux around para sport/event inclusion/exclusion and the volatility surrounding selection of classification categories jeopardizes CGA's long term planning around athlete development, a reality that is further exacerbated when resources and infrastructure are limited. Persistent selection of high resource sports like that of wheelchair racing and para triathlon is highly problematic for developing nations. The expense associated with the basic necessities for participation is not only prohibitive but likely negatively impacts a developing nation or territory's ability to be competitive in a field of para sport competitors from predominantly developed CW locations. Yet, it is clear why these events are chosen because they do not disrupt the ableist organizational structure of the Games and require little expectations to reducing non-disabled participants to manage the size. These justifications are built of fundamental ableist logics that deny the rights of persons with disabilities.

The effects of ableist structures and practices associated with classification are experienced by the least powerful, those who occupy the “bottom rungs” of the social ladder, a most dismantling form of structural violence. Our research findings echo that of many (2, 3, 18, 29) whose work demonstrates that classification with the current practices is fundamentally a tool to eliminate competition opportunities for athletes with higher needs, in favour of more abled-disabled athletes (2, 3). Decisions to include classifications at CG that include athletes with less impaired bodies and to conversely exclude athletes with greater impairment, is highly problematic to CGF's explicit wish to “grow” representation in para sport at CG from developing CW nations/territories and poses an existential threat to the realization of the values of “humanity, destiny and equality”.

### Classification as a catalyst for inclusion

The World Health Organization (43) estimates that 15% of the global population lives with disability. At Commonwealth Games XXI and XXII respectively, 5.7% and 7.6% of the athlete delegation competed in the para sport program. The research of Quinn, Misener and Howe (3) found the inclusion of such a relatively small number of para sport athletes marginalized their value as athletes and undermined the credibility of high-performance para sport. By stabilizing classification volatility, classification can be used as a catalyst for para sport participation, high-performance development, and “getting to the Games” for athletes from lower resource regions of the Commonwealth. Decisions to eliminate classification volatility, by CGF and future CG organizing committees, also provides a path forward to an authentically integrated CG, where para sport athlete comprises 15% of the athlete delegation. Commitment to the inclusion of classification categories that foreground, rather than minimize bodily difference provides an opportunity for CGF to capitalize that which is distinct and different about Commonwealth Games, simultaneously resisting the ableization of para sport, and realizing the values of the Commonwealth movement in and through sport.

## Final thoughts: rights, classification and participation

As we conclude, we reiterate that there is much for future researchers to explore and unpack with respect to the work that we have reviewed. We carefully celebrate the gender parity and the inclusion of para sport athletes on the teams of eleven additional CW nations/territories in Birmingham in 2022. We anticipate the announcement of events and sport classifications for the next iteration of Commonwealth Games, to take place in Victoria Australia in 2026 to demonstrate an increased commitment to para sport. Our review however illustrates that throughout the history of the integrated Commonwealth Games, classification for inclusion at CG moves towards the center, where classification categories include athletes with similar impairments. We suggest the integration of some of the athletes from the highly diverse para sport community are the “more abled-disabled” (2). These athletes may be more relatable for the sport spectator/consumer, and less challenging for the media to construct as high-performance athletes. Decisions to include those with minimal impairment makes difference invisible on the field of play, simplifying the “selling” of sport. Ableization of para sport at CG eliminates the need to inform let alone educate fans of CW sport about sporting lives with impairment and the impact of classification on competition. The repercussion is non-understandings of the impact of impairment on athletic ability and a missed opportunity to celebrate human difference, and the abilities of the high-performance body with impairment. Historically and ultimately, classification within the context of Commonwealth Games has evolved to favour the most able, pushing out or rather “classifying out”, athletes with higher support needs and greater impairment. We conclude with a loud and persistent call for change, a transformational change to the way in which classification has historically been used at CG and embrace classification as a tool to broaden representation across the CW, for developing CW nations and athletes with greater impairment. Classification could be a catalyst for para sport participation across the Commonwealth and at future CG if it becomes more accessible. In creating an environment where athletes with impairment are truly valued throughout the CW, CGF is then positioned to realize the values of “equity, destiny and humanity”, to facilitate access to the rights of CW citizens who live with impairment to sport participation and continue to lead in the pursuit of authentically integrated, high-performance sport.
